# Novel *NUTM1* Fusions in Relapsed Acute Myeloid Leukemia: Expanding the Genetic and Clinical Landscape

**DOI:** 10.3390/ijms262311676

**Published:** 2025-12-02

**Authors:** Parastou Tizro, Lisa Chang, Amandeep Salhotra, Javier Arias-Stella, Milhan Telatar, Vanina Tomasian, Karl Gaal, Joo Song, Lorinda Soma, Sandra Fuentes, Lino Garcia, Fei Fei, Anamaria Munteanu, Guido Marcucci, Michelle Afkhami

**Affiliations:** 1 Pathology, Molecular Pathology and Therapy Biomarkers Division, City of Hope Comprehensive Cancer Center, 1500 East Duarte Road, Duarte, CA 91010, USA; ptizro@coh.org (P.T.);; 2 Pathology, Hematopathology, City of Hope Comprehensive Cancer Center, 1500 East Duarte Road, Duarte, CA 91010, USA; 3 Hematology and Transplant, Leukemia Division, City of Hope Comprehensive Cancer Center, Duarte, CA 91010, USA; 4 Cytogenetics/Cytogenomics, City of Hope Comprehensive Cancer Center, Duarte, CA 91010, USA; 5 Pathology, Hematopathology, University of California, Los Angeles, Los Angeles, CA 90095, USA

**Keywords:** *NUTM1* fusion, acute myeloid leukemia, monocytic differentiation

## Abstract

Gene fusions involving *NUTM1* have been increasingly recognized in hematologic malignancies, though their role in acute myeloid leukemia (AML) remains poorly understood. We retrospectively analyzed 565 unique AML patients with reported fusion results who underwent comprehensive next-generation sequencing (NGS) between March 2022 and December 2023. Among them, three novel in-frame *NUTM1* fusion transcripts, *LARP1::NUTM1*, *ARHGAP15::NUTM1,* and *GABPB1::NUTM1*, were identified in three relapsed or refractory AML cases, all with monocytic differentiation. Ancillary studies included flow cytometry, cytogenetics, FISH, and comprehensive mutational profiling. All three patients eventually relapsed and succumbed to their disease, despite initial responses in one case. Each case also harbored co-occurring mutations associated with adverse prognosis, such as *BCOR, ASXL1,* and *RUNX1*. These findings suggest *NUTM1* fusions in AML could represent a distinct molecular subset with potentially poor prognosis, warranting further functional and clinical investigation to clarify their biological and therapeutic significance.

## 1. Introduction

Gene fusions are frequent in acute myeloid leukemia (AML), with their oncogenic potential and prognostic impact varying widely [1]. Advances in RNA sequencing have led to the discovery of several novel fusions, although their clinical significance remains unknown and awaits further investigation.

The NUT midline carcinoma family member 1 (*NUTM1*) gene is a fusion partner of growing interest in hematological disorders. Located on chromosome 15, *NUTM1* encodes the nuclear protein in testis (NUT) and is normally expressed in the testis and ciliary ganglion [2]. These fusions are a defining event in NUT carcinomas and are associated with poor prognosis [2,3].

Although rare, rearrangements of *NUTM1* have been identified in hematologic malignancies, primarily in *KMT2A*-wildtype infant and pediatric B-lymphoblastic leukemia (B-ALL) [4]. *NUTM1* fusions are extremely rare in AML, but emerging evidence suggests they may have clinical significance.

*TIPIN::NUTM1* fusion was first identified in a study that conducted whole-transcriptome sequencing of 572 AML patients, representing a novel fusion discovery [5]. Recently, an *AVEN::NUTM1* fusion was reported in a relapsed AML case, which has been shown to drive myeloid leukemia in mouse models [6].

Here, we present three additional cases of relapsed AML, each harboring a novel in-frame *NUTM1* fusion. These discoveries broaden our understanding of the genetic landscape of AML as well as the potential clinical and prognostic implications of these alterations in this cancer.

## 2. Case Description

Case 1 involves a 65-year-old male who presented with pancytopenia in September 2020. A BM biopsy (BMBx) confirmed AML with *IDH2* mutation detected by NGS at an outside hospital. The patient entered remission after receiving induction chemotherapy with a 7 + 3 regimen (cytarabine + idarubicin) and was placed on enasidenib maintenance. He relapsed in January 2022, and BMBx revealed 20% myeloblasts. Cytogenetic studies showed a complex karyotype including partial deletions and additions involving chromosome 15. Molecular testing identified *DNMT3A*, *IDH2*, and *BCOR* mutations. He received salvage chemotherapy with fludarabine, cytarabine, and idarubicin (FLAG-Ida) but had refractory AML, and a BMBx in April 2022 showed persistent disease. Cytogenetic analysis demonstrated t(9;15)(q34;q15) in 10/20 metaphase cells and one non-clonal cell showing t(5;15)(q33;q11.2). He received salvage chemotherapy with fludarabine, cytarabine, etoposide, and venetoclax. Post-therapy BMBx showed morphologic complete remission (CR)-2 and no evidence of residual leukemia according to measurable residual disease (MRD) flow cytometry (FC); cytogenetic studies were also negative. The *IDH2* mutation persisted at 16% variant allele frequency (VAF).

Two months later, the patient relapsed, characterized by an abnormal myelomonocytic immature population. HopeSeqHC identified *ASXL2*, *BCOR*, *DNMT3A*, and *IDH2* mutations and a *LARP1::NUTM1* fusion involving exon 1 of *LARP1* fused to exon 2 of *NUTM1* (breakpoints at chr5:154135753 and chr15:34640170; GRCh38). The karyotype demonstrated t(5;15)(q33;q11.2). The patient started on salvage chemotherapy with decitabine, venetoclax, and enasidenib. Day-28 BMBx showed MRD-negative CR-3. Later, he underwent an allogeneic hematopoietic stem cell transplant (HSCT). BMBx on day 30 post-transplant showed remission, and *IDH2* mutation analysis was negative.

However, day +100 BM revealed relapsed disease, with 38% blasts showing monocytic differentiation. Cytogenetic analysis again detected t(5;15)(q33;q11.2). Molecular testing of PB showed *ASXL2*, *BCOR*, *DNMT3A*, *IDH2*, and *NSD1* mutations and the previously identified *LARP1::NUTM1* fusion, with elevated *CDK6*, *FLT3*, and *LMO2* expression. The patient passed away in January 2023.

Case 2: A 67-year-old male who was incidentally found to have pancytopenia. Further evaluation at an outside facility in early 2022 revealed 17% blasts in the PB. A BMBx showed 90% blasts with a myelomonocytic phenotype according to flow cytometry. Molecular and fluorescence in situ hybridization (FISH) studies for AML were negative. The patient received induction therapy (7 + 3 regimen), and a post-induction BMBx revealed 45% blasts with a normal karyotype. He subsequently received salvage therapy using FLAG-Ida in mid-April, and a day-18 BMBx demonstrated CR-1. He then received consolidation with high-dose cytarabine.

However, the patient relapsed one month later, and FISH studies were again negative. Azacitidine and venetoclax reinduction was initiated. A follow-up BMBx revealed persistent disease, with 35% blasts showing monocytic differentiation. Notably, the karyotype showed the presence of t(2;15)(q23;q15) in 9/20 cells. NGS of repeated in-house BMBx detected *ASXL1*, *RUNX1*, and *TET2* mutations and a novel *ARHGAP15::NUTM1* fusion, involving exon 5 of *ARHGAP15* fused to exon 1 of *NUTM1* (breakpoints at chr2:143986237 and chr15:34638143; GRCh38). A limited karyotype study of 11 mitotic cells was normal. The patient was enrolled in a clinical trial with decitabine, venetoclax, and navitoclax (NCT05222984). Subsequent BMBx showed persistent disease, and no mitotic cells were available for cytogenetic analysis. The patient failed to respond to treatment and passed away.

Case 3 involves a 72-year-old male, first diagnosed with AML in 2011, who presented with fever, fatigue, and abdominal pain. Cytogenetic analysis revealed an isochromosome 7p along with two distinct clones harboring a t(9;11) translocation and a t(4;12) translocation. Additionally, a non-clonal abnormality involving t(15;16) was observed. FISH revealed a *KMT2A* rearrangement, and a rapid AML panel was negative. The patient received induction (7 + 3 regimen), followed by high-dose cytarabine, but failed to achieve remission, and was also refractory to decitabine and DNA methyltransferase inhibitors received through a clinical trial. He was subsequently treated with FLAG and etoposide and achieved CR-1. In August 2012, the patient underwent an allogenic HSCT, leading to prolonged remission.

**Figure 1:**
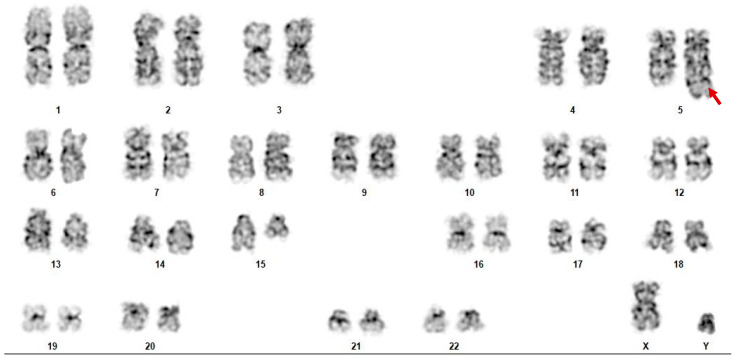
Karyotype analysis in a representative patient (case 1) shows a balanced translocation involving chromosomes 5 and 15. The image demonstrates a rearrangement involving chromosome 5q33.2 (*LARP1* locus) and chromosome 15q14 (*NUTM1* locus), compatible with a *LARP1::NUTM1* fusion. Red arrow is marking chromosomes 5 and 15, which participate in the translocation event.

During a follow-up check on April 2023, he was found to be thrombocytopenic. A BMBx revealed relapsed AML, with 80–85% blasts with monocytic differentiation. A cytogenetic analysis showed a recipient stemline in 16/20 cells, and FISH was negative for *KMT2A* rearrangement. NGS identified *AMER1*, *BCOR*, *DNMT3A*, *GATA2*, *IDH1,* and *PHF6* mutations; *NF1* loss; elevated *FLT3* and *LMO2* expression; and *GABPB1::NUTM1* fusion, characterized by fusion of *GABPB1* exon 1 (5′ UTR) with *NUTM1* exon 2 (breakpoints mapped to chr15:50647182 and chr15:34640170; GRCh38). The patient resumed treatment with azacitidine and venetoclax in June 2023 and achieved CR-2 a month later, with prolonged remission thereafter. On March 2024, he developed severe back pain, and imaging revealed a paraspinal mass. In April 2024, a cerebral spinal fluid (CSF) sample showed 91% blasts, with an abnormal myelomonocytic phenotype according to flow cytometry. A concurrent BMBx showed no morphologic evidence of leukemia, corroborating an extramedullary relapse. The patient continued to experience significant paralysis and deconditioning and passed away on May 2024. Table 1 summarizes the immunophenotypic, molecular, and genetic findings (representative case illustrated in Figure 1).

## 3. Discussion

All three patients had relapsed or refractory AML and acquired novel *NUTM1* fusions. *NUTM1* fusions appear to represent an adverse prognostic event in AML, as all patients relapsed post-transplant and ultimately died, although one case (Case 3) achieved prolonged remission.

Interestingly, in pediatric B-ALL, *NUTM1* fusions have been associated with favorable outcomes [4]; however, the fusion partners described including *ACIN1*, *BRD9*, and *CUX1* being the most frequent, do not overlap with those identified in our patients or in previously reported AML cases [5]. Our findings add to the growing complexity of *NUTM1* rearrangement’s role in hematologic malignancies and raise the possibility that the nature of the *NUTM1* fusion partner or disease biology may influence the treatment response and clinical outcomes.

The fusion partner in Case 1 was *LARP1*. *LARP1*, located on chromosome 5, encodes La-related protein 1, an RNA-binding protein associated with the mammalian target of rapamycin (mTOR) signaling pathway. The mTOR pathway plays a critical role in regulating cell proliferation, growth, migration, and apoptosis, and its dysregulation has been implicated in various cancers, including hematologic malignancies [7]. Specifically, activation of the mTORC1 complex has been shown to promote tumor progression in mouse models, and its overexpression is associated with poor prognosis in ovarian, colorectal, and prostate cancers. A *LARP1::MRPL22* fusion has been reported to be recurrently expressed in B-cell non-Hodgkin lymphoma; however, its clinical significance remains unclear [8,9].

The fusion partner in Case 2 was *ARHGAP15*, which is located on chromosome 2 and encodes Rho GTPase-activating protein. This protein is involved in the inactivation of Rac1 (Ras-related C3 botulinum toxin substrate 1). Abnormal expression of *ARHGAP15* has been reported in human gliomas, where it is associated with more aggressive tumor phenotypes [10]. In contrast, another study found that *ARHGAP15* immunoreactivity correlated with improved prognosis and a lower risk of recurrence in breast carcinoma tissues, possibly through Rac1 inactivation [10]. To our knowledge, *ARHGAP15* fusions have not been previously reported in hematologic disorders [11].

**Figure 2:**
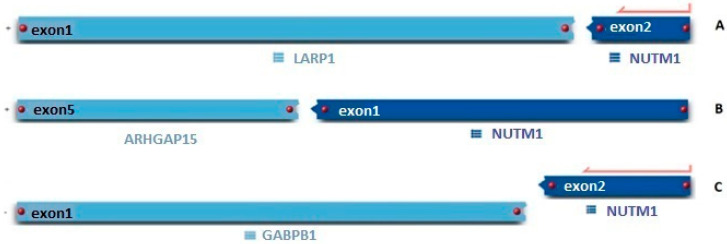
(**A**) RNA sequencing detected the in-frame novel fusion of exon 1 of *LARP1* to exon 2 of *NUTM1* at chr5:154135753,chr15:34640170; GRCh38. (**B**) RNA sequencing detected an in-frame novel fusion of exon 5 of *ARHGAP15* to exon 1 of *NUTM1* at chr2:143986237,chr15:34638143; GRCh38. (**C**) RNA sequencing detected novel fusion of exon 1 (5′ UTR) of *GABPB1* to exon 2 of *NUTM1* at chr15:50647182,chr15:34640170; GRCh38.

In Case 3, the fusion partner was *GABPB1*, which encodes the GA-binding protein transcription factor, beta subunit 1. Located on chromosome 15, *GABPB1* plays a role in the activation of cytochrome oxidase expression and in the nuclear regulation of mitochondrial function [12]. Altered *GABPB1* expression has been shown to disrupt myeloid differentiation in CD34^+^ hematopoietic stem cells, potentially contributing to leukemogenesis (Figure 2) [13].

All three patients described, despite having different fusion partners, demonstrated relapsing/refractory AML after transplant, and all had dismal outcomes. In addition, a previously reported case with a novel *AVEN::NUTM1* fusion relapsed post-transplant [6].

While our cases harbored mutations associated with poor prognosis, such as in *BCOR*, *ASXL1*, and *RUNX1*, according to European LeukemiaNet (ELN) and World Health Organization (WHO) recommendations, the short relapse time and dismal outcome might be attributable to *NUTM1* fusions or their association with these adverse alterations [1,14]. Due to the limited sample size, the prognostic significance of *NUTM1* fusions in AML has not been fully established in this study.

Interestingly, all cases demonstrated monocytic features, as in the previously reported *AVEN::NUTM1* AML case [6], suggesting common morphologic and immunophenotypic features in these leukemias.

**Figure 3:**
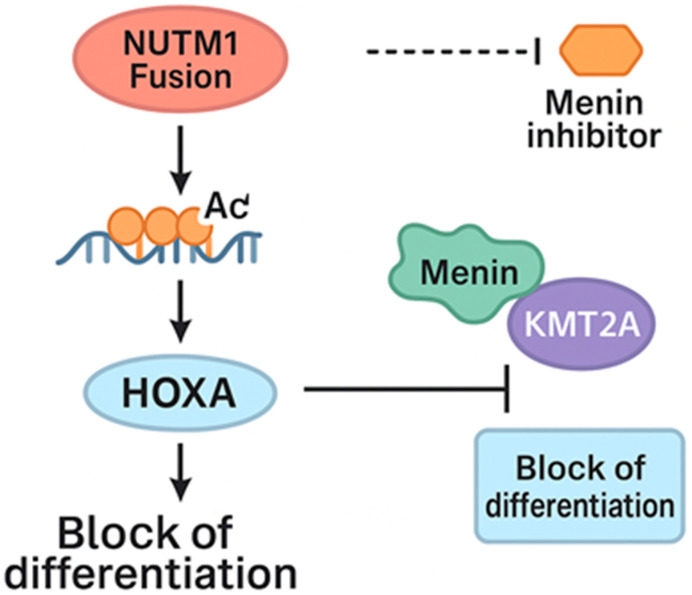
The schematic depicts how *NUTM1* fusion proteins promote chromatin hyperacetylation and mega domain formation, leading to transcriptional activation of the *HOXA* gene cluster. Elevated *HOXA* expression maintains a block of differentiation, contributing to leukemogenesis. The Menin–*KMT2A* complex further supports *HOXA* transcriptional activity. Inhibition of Menin disrupts this interaction, resulting in downregulation of *HOXA* expression and restoration of differentiation, suggesting a mechanistic rationale for the sensitivity of *NUTM1*-rearranged malignancies to Menin inhibitors.

Furthermore, in B-ALL, certain *NUTM1* rearrangements have been found to correlate with Homeobox A9 (HOXA9) expression [4]. Here, elevated *LMO2* and *FLT3* expression, both known to be co-upregulated in human and murine models of HOX-driven AML [14], was observed in two out of the three patients, further supporting this association (Figure 3). The upregulation of HOXA genes has also been reported to confer sensitivity to Menin inhibitors in certain leukemias [4]. In addition, the *AVEN::NUTM1* fusion has been shown to confer sensitivity to histone deacetylase (HDAC) inhibitors in preclinical studies [6]. These findings underscore possible therapeutic implications for *NUTM1* fusions and warrant further investigation.

## 4. Materials and Methods

We performed a retrospective review of AML patients who underwent next-generation sequencing (NGS) at City of Hope between March 2022 and December 2023. Bone marrow and peripheral blood specimens were analyzed using the HopeSeq Heme Comprehensive (HopeSeqHC) panel, which integrates DNA (523 genes) and RNA (165 genes) sequencing. The DNA component utilized the Illumina TruSight Oncology 500 (TSO500) assay (Illumina Inc., San Diego, CA, USA; version 2.5.2) to detect single-nucleotide variants, insertions/deletions, copy-number alterations, and splice-site variants. The RNA component employed Archer’s Anchored Multiplex PCR (AMP™)–based custom NGS assay (Integrated DNA Technologies, Coralville, IA, USA) on the Thermo Fisher Ion GeneStudio™ S5 system (Thermo Fisher Scientific, Waltham, MA, USA).

Bioinformatic analysis was performed using ArcherDX software (version 7.3.2, ArcherDX, Boulder, CO, USA). All fusion calls were confirmed through manual review. Conventional karyotyping and fluorescence in situ hybridization (FISH) were performed following standard laboratory protocols.

**Figure 4:**
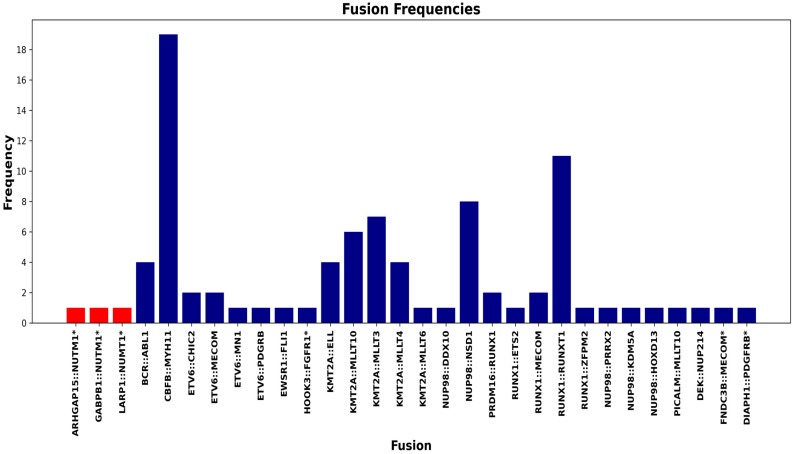
Bar chart shows the frequency of gene fusions observed in the dataset. Each bar represents a distinct gene fusion event, with the height corresponding to its frequency. In total, 88 patients were positive for at least 1 fusion. Only 1/88 patients were positive for 2 fusions. The asterisk (*) marks fusions that had not been reported in the literature or publicly available databases as of our last search on 3 July 2025. Red-colored labels indicate the three novel *NUTM1* fusions identified in our study.

In total, 89 fusions were detected among 88 patients, including three novel *NUTM1* rearrangements (Figure 4). Karyotype images were generated using CytoVision (Leica Biosystems, Buffalo Grove, IL, USA) and GenASIs (Applied Spectral Imaging, Carlsbad, CA, USA; version 8.2.2). Fusion schematics were created using Archer Analysis software (ArcherDX, Boulder, CO, USA; version 7.3.2). Additional figure preparation and visualization were performed using BioRender.com and Python’s matplotlib package (Python Software, https://matplotlib.org).

## Figures and Tables

**Table 1:** Summary of the immunophenotypic, molecular, and genetic findings for all 3 cases.

Case Number	Test	At Time of Diagnosis	Relapsed/Refractory Disease (Pre-Transplant)	Relapsed/Refractory Disease (Post-Transplant)
Case 1	Cytogenetics	45~46, X, −Y, add(9)(q34), del(15)(q11.2)[cp6]/46, XY, del(15)(q22q24), del(20)(q11.2q13.1)[cp5]/46, XY, add(15)(q24)[2]/46, XY[7]	46, XY, t(3;11;6)(p21;p15;q23), t(5;15)(q33;q11.2)[19] 46, XY[3]	46, XY, t(3;11;6)(p21;p15;q23), t(5;15)(q33;q11.2)[18] 46, XX[2]
	RNA-seq		*LARP1::NUTM1*	*LARP1::NUTM1* Elevated expression: CDK6, FLT3, LMO2
	DNA-seq	*DNMT3A* (D845Afs*8), *IDH2* (R172K), and *BCOR* (S1263*)	*ASXL2* (K873Nfs*6), *BCOR* (S1297*), *DNMT3A* (D845Afs*8), *IDH2* (R172K)	*ASXL2* (K873fs*6), *BCOR* (S1297*), *DNMT3A* (D845fs*8), *IDH2* (R172K), *NSD1* (V1016fs*27)
	Flow Cytometry		Expanded (40%) abnormal CD34-negative immature “monocytic” population expressing CD4, CD11c, CD13 (dim), CD15 (strong), CD33 (strong), CD38, CD123, and HLA-DR. This population is negative for CD117 and CD34. Proportion of 12.3% abnormal myeloid blasts expressing CD13 (subset increased), CD33, CD34, CD38 (slightly decreased), CD45 (dim), CD117, and CD123 (dim)	Expanded abnormal myeloid blast population detected (~38% of total analyzed white blood cells) expressing CD4 (partial), CD7, CD9 (partial), CD11c (partial), CD13 (dim), CD15 (subset), CD33, CD34, CD38 (decreased), CD45 (dim), CD58, CD117, CD123 (moderate), and HLA-DR
Case 2	Cytogenetics	Normal	t(2;15) (q23;q15)[9]/46, XY[11]	Limited study with normal karyotype
	RNA-seq			*ARHGAP15::NUTM1*
	DNA-seq	Negative for tested genes (*CEBPA*, *IDH1/IDH2*, *FLT3-ITD*, *FLT3-TKD*, *KIT*, *NPM1*)		*ASXL1* (G646fs*12), *RUNX1* (R169fs*44), *TET2* (Q1942*)
	Flow Cytometry	Flow cytometry revealed increased monocytes (46%) with aberrant CD56 expression along with 10% CD34-positive myeloblasts. Blasts expressed CD7, CD13, CD33, CD34, CD38, CD117, and HLA-DR	Increased immature myelomonocytic population (24%)	Abnormal monocytic cell population (>90% of total analyzed cells) expressing CD4, CD7 (small subset), CD9 (partial), CD11b, CD11c, CD13 (decreased), CD14, CD15, CD16 (partial), CD33, CD38, CD45 (bright, monocytic gate), CD56 (minor subset), CD64, CD123 (moderate), and HLA-DR (partial)
Case 3	Cytogenetics	46, XY, i(7)(p10), t(9;11)(p22;q23)[6] 46, XY, i(7)(p10), t(4;12)(q12;p13)[5] Non-clonal aberration of clone 1: t(15;16)(q15;q22) FISH studies: 11.7% *KMT2A* translocation		46, XY, i(7)(p10), t(4;21)(q12;q22), del(13)(q14q22)[16] 46, XX[4]
	RNA-seq			*GABPB1::NUTM1* Elevated expression: *FLT3* and *LMO2*
	DNA-seq	Negative		*AMER1* (R1049*), *BCOR* (K395fs*47), *DNMT3A* (R882H), *GATA2* (R362Q), *IDH1* (R132C), *PHF6* (Q37*), *NF1* loss
	Flow Cytometry	Flow cytometric analysis of the “blast” gate showed an increased population of myeloid blasts positive for HLA-DR, CD45, CD15, dim CD13, CD11b, and CD64, consistent with persistent acute myeloid leukemia		Expanded population of abnormal myelomonocytic blasts (67.5%) expressing CD4 (subset), CD9 (subset), CD11b (subset, dim), CD13 (increased), CD33 (dim), CD34, CD38, CD58 (dim), CD64, CD117, CD123 (moderate), HLA-DR, and MPO (subset)

## Data Availability

The original contributions presented in this study are included in the article. Further inquiries can be directed to the corresponding author.

## Abbreviations

The following abbreviations are used in this manuscript:

AML	Acute Myeloid Leukemia
NUTM1	NUT Midline Carcinoma Family Member 1
BMBx	Bone Marrow Biopsy
HSCT	Hematopoietic Stem Cell Transplant
FISH	Fluorescence In Situ Hybridization
HOXA9	Homeobox A9
LMO2	LIM Domain Only 2
ELN	European Leukemia Network
WHO	World Health Organization
MRD	Measurable Residual Disease
